# Routine biochemical indices for hepatometabolic stratification across the dysglycaemia spectrum: insulin resistance, fibrosis scores, and machine-learning integration

**DOI:** 10.1515/almed-2026-0066

**Published:** 2026-07-14

**Authors:** Pedro Villafruela-Rodríguez-Manzaneque, Jordi Tortosa-Carreres, Antonio Sierra-Rivera, Manuela Morales-Garcés, Laura Sahuquillo-Frias, Goitzane Marcaida-Benito

**Affiliations:** Laboratory Department, Consorci Hospital General Universitari de València, València, Spain; Laboratory Department, Hospital Universitari i Politècnic la Fe, València, Spain

**Keywords:** diabetes mellitus, insulin resistance, biochemical markers, hepatic fibrosis, metabolic alterations

## Abstract

**Objectives:**

To evaluate the behaviour of insulin resistance indices and non-invasive liver fibrosis scores across the dysglycaemia spectrum using routine laboratory data.

**Methods:**

This retrospective observational study included 1,949 outpatients with complete biochemical profiles. Patients were classified into five groups according to fasting plasma glucose and HbA_1c_: group A (normoglycaemia), group B (isolated impaired fasting glucose), group C (isolated elevated HbA_1c_), group D (combined impairment), and group E (type 2 diabetes mellitus, T2DM). Insulin resistance indices (HOMA-IR, HOMA-β, QUICKI, TyG), liver fibrosis scores (FIB-4, APRI, m-APRI, Forns), and inflammatory markers (CRP, ferritin) were compared across groups. Discriminative performance was assessed using ROC analysis for the identification of T2DM (group E vs. groups A–D) and early dysglycaemic impairment (group D vs. group A). Support vector machine (SVM) models integrating the best-performing indices were constructed with adjustment for age and sex.

**Results:**

Insulin resistance indices, fibrosis scores, and CRP showed progressive deterioration across worsening glycaemic stages (p<0.001). TyG showed the highest individual discriminative performance for identifying T2DM (AUC=0.90), whereas QUICKI performed best for detecting combined early glycaemic impairment (AUC=0.78). Among fibrosis scores, the Forns index showed the strongest discriminative capacity (AUC=0.67–0.74) and the broadest correlations with metabolic and inflammatory parameters. SVM models integrating insulin resistance and fibrosis indices improved classification performance compared with individual markers alone (AUC=0.87–0.93).

**Conclusions:**

TyG and the Forns index support laboratory-based hepatometabolic stratification across the dysglycaemia spectrum using routine analytical data.

## Introduction

Type 2 diabetes mellitus (T2DM) is a chronic metabolic disorder whose global burden is rapidly increasing, with projections estimating that it will affect more than 850 million people by 2050 [1]. Its pathophysiology is primarily driven by insulin resistance, which impairs cellular glucose uptake and promotes hepatic gluconeogenesis and glycogenolysis, resulting in chronic hyperglycaemia [2], 3]. The progression to overt T2DM does not occur abruptly but develops through an intermediate stage known as prediabetes, defined by the American Diabetes Association (ADA), in which clinically relevant metabolic alterations are already present despite the absence of a formal diagnosis of diabetes [1].

Insulin resistance is closely associated with disturbances in lipid metabolism, including elevated triglycerides, predominance of low-density lipoprotein cholesterol (LDL-C) particles, and reduced high-density lipoprotein cholesterol (HDL-C) concentrations, forming an atherogenic profile that increases the risk of atherosclerotic cardiovascular disease, the leading cause of morbidity and mortality in T2DM [4], 5]. In parallel, insulin resistance enhances peripheral lipolysis and hepatic free fatty acid influx, promoting hepatic steatosis and contributing to the development of metabolic dysfunction-associated steatotic liver disease (MASLD), previously termed non-alcoholic fatty liver disease (NAFLD) [6], 7]. MASLD may progress to metabolic dysfunction-associated steatohepatitis (MASH), fibrosis, cirrhosis, and hepatocellular carcinoma [7], 8], affecting 49–62 % of patients with T2DM, with 2.8–5.6 % advancing to fibrosis as disease severity increases [9]. MASLD is also associated with greater metabolic impairment, visceral adiposity, systemic inflammation, and increased mortality, reinforcing its role within the metabolic continuum linked to insulin resistance [10], 11].

Several surrogate indices quantify insulin resistance in clinical practice, avoiding complex methods such as the hyperinsulinaemic–euglycaemic clamp (HEC), which remains the reference technique but is impractical for routine use [3]. Among indices based on fasting glucose and insulin, the Homeostatic Model Assessment of Insulin Resistance (HOMA-IR) is the most widely used and strongly correlated with HEC [3], 12], whereas the Homeostatic Model Assessment of β-cell function (HOMA-β) estimates pancreatic β-cell function [3], 13]. The Quantitative Insulin Sensitivity Check Index (QUICKI) also correlates closely with HEC, particularly in obese populations [3], 14], 15]. Simpler indices not requiring insulin measurements include the triglyceride–glucose (TyG) index, associated with HOMA-IR and predictive of metabolic syndrome and cardiovascular disease [12], 16], 17], and the triglycerides/high-density lipoprotein cholesterol (TG/HDL-C) ratio, a lipid-derived marker of insulin resistance with predictive value for metabolic syndrome, T2DM, and cardiovascular events [17], 18].

Hepatic steatosis and fibrosis can be evaluated using clinical assessment, imaging techniques, biopsy, and laboratory-derived indices. Although liver biopsy remains the reference standard for fibrosis staging, its invasive nature limits routine use, making non-invasive alternatives such as transient elastography (Fibroscan^®^) and laboratory-based fibrosis scores increasingly relevant [19]. Serum hepatic enzymes – including alanine aminotransferase (ALT), aspartate aminotransferase (AST), and gamma-glutamyl transferase (GGT) – reflect hepatocellular injury and inflammatory activity, but up to 80 % of MASLD patients present normal aminotransferase levels, limiting their utility for fibrosis staging [6], 7].

Composite fibrosis scores derived from routine analytical parameters provide a practical alternative for estimating fibrosis risk without biopsy. These indices combine age, transaminases, platelet count, albumin, and lipid-related variables to estimate the probability of underlying hepatic fibrosis using routinely available laboratory data. [19]. The most widely used include the Fibrosis-4 index (FIB-4), the AST-to-Platelet Ratio Index (APRI) and its modified version (m-APRI), and the Forns index. FIB-4 shows high negative predictive value for detecting moderate-to-advanced fibrosis and cirrhosis and is recommended in MASLD by guidelines such as those of the American Association for the Study of Liver Diseases (AASLD) [6], 20]. APRI is based on AST and platelet count, whereas m-APRI incorporates albumin and age to improve performance in metabolic liver disease settings such as MASLD [6], 21]. The Forns index includes age, platelet count, GGT, and total cholesterol and has also demonstrated usefulness in MASLD populations [6], 7].

Furthermore, systemic inflammation represents an additional component in the progression of MASLD and in the pathophysiology of T2DM and can be monitored using markers such as C-reactive protein (CRP) or serum ferritin.

Although surrogate indices of insulin resistance and laboratory-derived fibrosis scores are widely used individually, their combined behaviour across the dysglycaemia spectrum remains insufficiently characterized in routine clinical laboratory settings. In this context, the objective of this study was to analyse the behaviour of biochemical markers of insulin resistance, hepatic fibrosis, and inflammation across the dysglycaemia spectrum, evaluate their discriminative capacity between glycaemic stages, and explore whether their combination through machine learning models improves metabolic risk stratification in clinical practice.

## Materials and methods

### Study design

A single-centre retrospective observational study was conducted using data extracted from the Laboratory Information System (LIS) of the Valencia General University Hospital. The study period extended from May 1, 2010, to January 31, 2025.

### Inclusion and exclusion criteria

Patients aged ≥18 years from outpatient clinics and primary care settings were included if they had a complete biochemical profile allowing calculation of insulin resistance indices, liver fibrosis scores, and lipid-derived ratios. Required parameters included glucose, HbA_1c_, and insulin; ALT, AST, GGT, platelet count, and albumin; and triglycerides (TG), total cholesterol (TC), HDL-C, and LDL-C.

These data were used to calculate insulin resistance indices (HOMA-IR, HOMA-β, QUICKI, TyG), liver fibrosis scores (FIB-4, APRI, m-APRI, Forns), mean estimated glucose (MEG)–derived from HbA_1c_ using standard conversion formulas-, and lipid-derived ratios (non-HDL-C, TG/HDL-C, TC/HDL-C), all computed using established formulas described in the literature [6], 19], 22].

Patients were excluded if they were younger than 18 years, pregnant, hospitalized, or had active oncologic or rheumatologic disease, given the potential use of pharmacological treatments that may significantly alter the biochemical parameters assessed. Such treatments included systemic glucocorticoids, immunosuppressive agents, monoclonal antibodies, and cytotoxic therapies known to alter glucose metabolism, lipid profile, or liver enzymes. Exclusion of these patients minimized confounding due to treatment-related variations in glycaemic, lipid, and hepatic function parameters.

### Patient classification

Patients were classified into five groups based on fasting glucose and HbA_1c_: Group A (normoglycaemia, fasting glucose ≤100 mg/dL and HbA_1c_ <5.7 %), Group B (isolated impaired fasting glucose, fasting glucose 101–125 mg/dL and normal HbA_1c_), Group C (isolated elevated HbA_1c, _HbA_1c_ 5.7–6.4 % and normal fasting glucose), Group D (combined impairment, fasting glucose 101–125 mg/dL and HbA_1c_ 5.7–6.4 %), and Group E (T2DM, fasting glucose ≥126 mg/dL and/or HbA_1c_ ≥6.5 %). Demographic variables, including age and sex, were also recorded.

The diagnosis of T2DM was established according to the Standards of Care in Diabetes-2025 [1], including HbA_1c_ ≥6.5 %, fasting plasma glucose ≥126 mg/dL, 2-h glucose ≥200 mg/dL during oral glucose tolerance testing when available, or random glucose ≥200 mg/dL in the presence of hyperglycaemic symptoms. In the absence of a previous clinical diagnosis, confirmation required two abnormal results obtained either simultaneously or at different time points.

Primary discrimination analyses focused on comparisons between normoglycaemia and combined impairment (A vs. D), normoglycaemia and T2DM (A vs. E), and combined impairment and T2DM (D vs. E), representing clinically relevant transitions across the dysglycaemia spectrum.

### Laboratory procedures

Blood samples were obtained by venipuncture in serum gel-separator tubes, allowed to clot for 30 min at room temperature, and centrifuged at 2,000 *g* for 10 min. Serum was processed immediately without prolonged storage.

Biochemical parameters – including glucose, liver enzymes, albumin, ferritin, CRP, and lipid profile – were analysed on AU5800 analyzers; insulin on DxI800; platelet counts on DxH platforms (Beckman Coulter^®^); and HbA_1c_ on HLC-723 G (Tosoh^®^). Analytical methodologies are detailed in Supplementary Table 1.

The laboratory is accredited under UNE-EN ISO 15189:2013 (No. 633/LE1866), ensuring quality and reliability. Methodological or equipment changes during the study period were validated for analytical interchangeability according to ISO 15189 requirements, guaranteeing result comparability over time.

### Statistical analysis

All statistical analyses were performed using RStudio (version 4.2.3). Normality of continuous variables was assessed using the Shapiro–Wilk test. As none of the quantitative variables followed a normal distribution, non-parametric tests were used throughout. Comparisons between two groups were performed using the Mann–Whitney U test, and comparisons among multiple groups using the Kruskal–Wallis test with Bonferroni post-hoc correction. Quantitative variables are presented as median (interquartile range), and categorical variables as absolute frequencies and percentages. Statistical significance was set at p<0.05.

Comparisons included insulin resistance indices, liver fibrosis scores, and inflammatory biomarkers (CRP and ferritin) across glycaemic groups and between sexes within each group.

Spearman’s rank correlation coefficient was used to evaluate associations between quantitative variables, including age, biochemical parameters, platelet count, insulin resistance indices, fibrosis scores, and inflammatory biomarkers. Correlation matrices were generated using the corrplot package, and p-values were computed with the psych package.

Discriminative capacity of the evaluated indices was assessed using receiver operating characteristic (ROC) curve analyses for the clinically relevant pairwise comparisons A vs. D, A vs. E, and D vs. E. Indices showing the highest individual discriminative performance within the training dataset were selected as predictors for classification modelling. For each comparison, one insulin-based index and one glucose- or lipid-derived index were incorporated together with age and sex as adjustment variables.

Support vector machine (SVM) models were implemented using the e1071 package, incorporating age and sex as covariates. Prior to model training, the dataset was randomly partitioned into training (70 %) and validation (30 %) subsets using the createDataPartition function from the caret package, and quantitative predictors were z-standardized using training-set parameters. Variable selection based on AUC was performed exclusively within the training dataset to avoid information leakage.

Several kernel functions (linear, polynomial, radial, and sigmoid) were evaluated, and the model achieving the highest validation AUC was retained. Model performance was assessed using AUC, optimal threshold (Youden index), sensitivity, specificity, positive predictive value, negative predictive value, and overall accuracy. ROC analyses were performed using the pROC package, and graphical outputs were generated with ggplot2.

### Ethical statement

The study protocol was approved by the Institutional Research Ethics Committee of Valencia General University Hospital (Approval No. 48–2025). The requirement for written informed consent was waived due to the retrospective design and full anonymization of patient data. All procedures complied with the Declaration of Helsinki and applicable data protection regulations.

## Results

A total of 1,949 patients were included and distributed across clinical groups as follows: 653 (34 %) in Group A, 378 (19 %) in Group B, 305 (16 %) in Group C, 310 (16 %) in Group D, and 303 (15 %) in Group E. Supplementary Table 2 summarises the median and interquartile range for age and each analysed biomarker, stratified by clinical group. None of the quantitative variables followed a normal distribution (Shapiro–Wilk p<0.001).

### Correlation analysis

**Figure 1:**
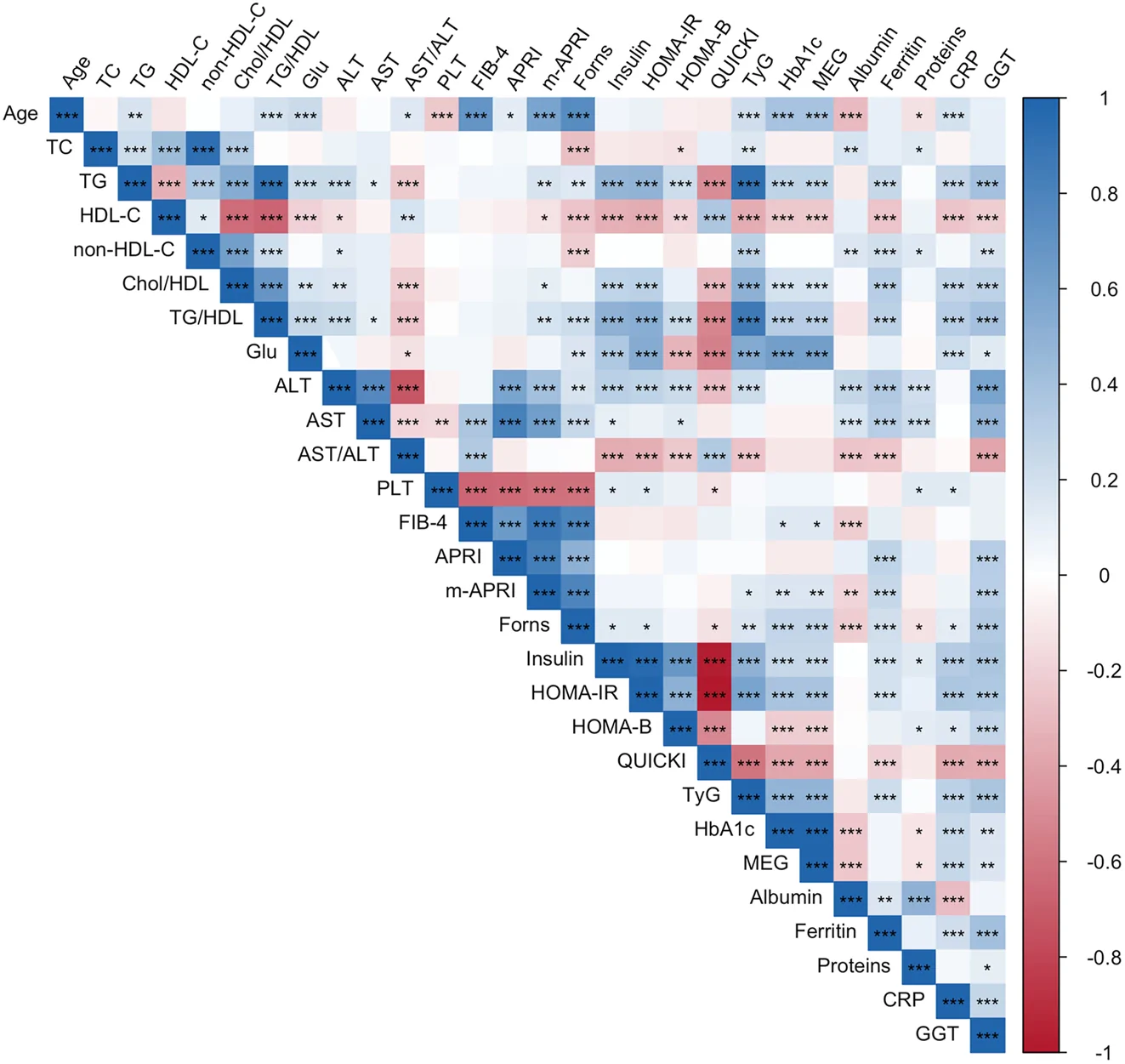
Correlation matrix of age, metabolic biomarkers, and derived clinical indices. Spearman correlation coefficients are shown for all pairwise associations. Absence of asterisks indicates non-significant correlations. *p<0.05; **p<0.01; ***p<0.001. Color scale represents Spearman’s rho correlation coefficients. ALT: alanine aminotransferase; AST: aspartate aminotransferase; Chol: total cholesterol; CRP: C-reactive protein; GGT: gamma-glutamyl transferase; Glu: fasting glucose; HbA_1c_: glycated haemoglobin; MEG: mean estimated glucose; TG: triglycerides.

Spearman correlation analyses between biochemical parameters, liver fibrosis indices, and insulin resistance markers are summarized in Figure 1, while the complete correlation matrix is provided in Supplementary File 1.

Regarding insulin resistance indices, all markers were significantly associated with fasting glucose, TG, HDL-C, ALT, AST/ALT ratio, HbA_1c_, MEG, and CRP. Platelet count showed weaker associations limited to HOMA-IR (ρ=0.14, p<0.05) and QUICKI (ρ=−0.14, p<0.05), whereas AST was only correlated with HOMA-β (ρ=0.15, p<0.05). TyG was additionally associated with age (ρ=0.24, p<0.001), non-HDL-C (ρ=0.27, p<0.001), and albumin (ρ=−0.13, p<0.05), suggesting a partially distinct correlation pattern compared with HOMA-derived indices.

Among biochemical parameters, all fibrosis indices were significantly associated with age, AST, GGT, platelet count, albumin, and ferritin, whereas none showed associations with CRP. All indices except FIB-4 were additionally correlated with ALT. Modest associations with triglycerides were observed for m-APRI (ρ=0.15, p<0.01) and Forns (ρ=0.18, p<0.01). Notably, the Forns index was the only fibrosis score correlated with lipid parameters (non-HDL-C: ρ=−0.16, p<0.01; LDL-C: ρ=−0.22, p<0.001; HDL-C: ρ=−0.22, p<0.001) and fasting plasma glucose (ρ=0.17, p<0.01).

Direct associations between fibrosis indices and insulin resistance markers were limited. Among them, only the Forns score showed modest correlations with HOMA-IR (ρ=0.15, p<0.01), QUICKI (p=−0.15, p<0.01), and TyG (ρ=0.12, p<0.01).

Inflammatory markers showed a differential pattern: ferritin correlated consistently with fibrosis indices, whereas CRP was associated only with insulin resistance markers and not with fibrosis scores (Figure 1).

### Group comparisons

Overall differences across glycaemic categories were observed for all evaluated fibrosis indices, insulin resistance indices, and inflammatory markers (Kruskal–Wallis test, p<0.001 for all variables). Post-hoc pairwise comparisons are detailed below.

#### Fibrosis indices

**Figure 2:**
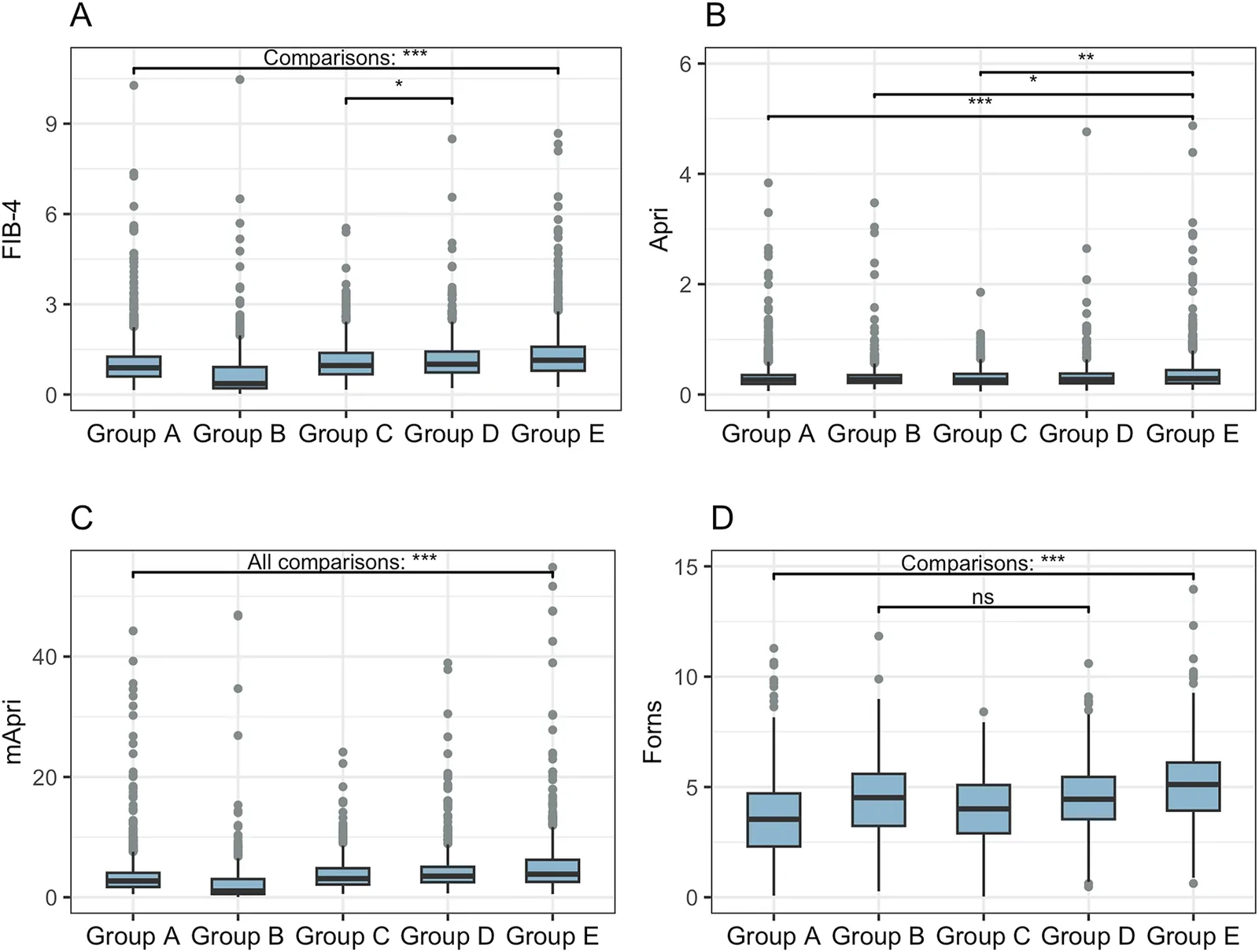
Distribution of liver fibrosis scores across study groups. Boxplots show the distribution of FIB-4 (A), APRI (B), modified APRI mAPRI (C), and forns index (D) across five groups. Boxes represent median and IQR; p-values correspond to Bonferroni-adjusted post-hoc comparisons after Kruskal–Wallis testing. ns=not significant; *p<0.05; **p<0.01; ***p<0.001.

FIB-4 values (Figure 2A) differed significantly across groups (p<0.001), except between Groups C and D, where differences were milder (p<0.05). Group B showed the lowest values, with a progressive increase from Groups C through E. APRI (Figure 2B) was significantly elevated in Group E compared with Groups A (p<0.001), B (p<0.05), and C (p<0.01). No significant differences were observed between Groups D and E. m-APRI (Figure 2C) showed highly significant differences across all group comparisons (p<0.001), with Group B presenting lower values than Group A and a rising trend from Group C to E. The Forns index (Figure 2D) also differed significantly across groups (p<0.001), except between Groups B and D. Group B had higher values than Group A, followed by a progressive increase from Groups C to E.

#### Insulin resistance indices

**Figure 3:**
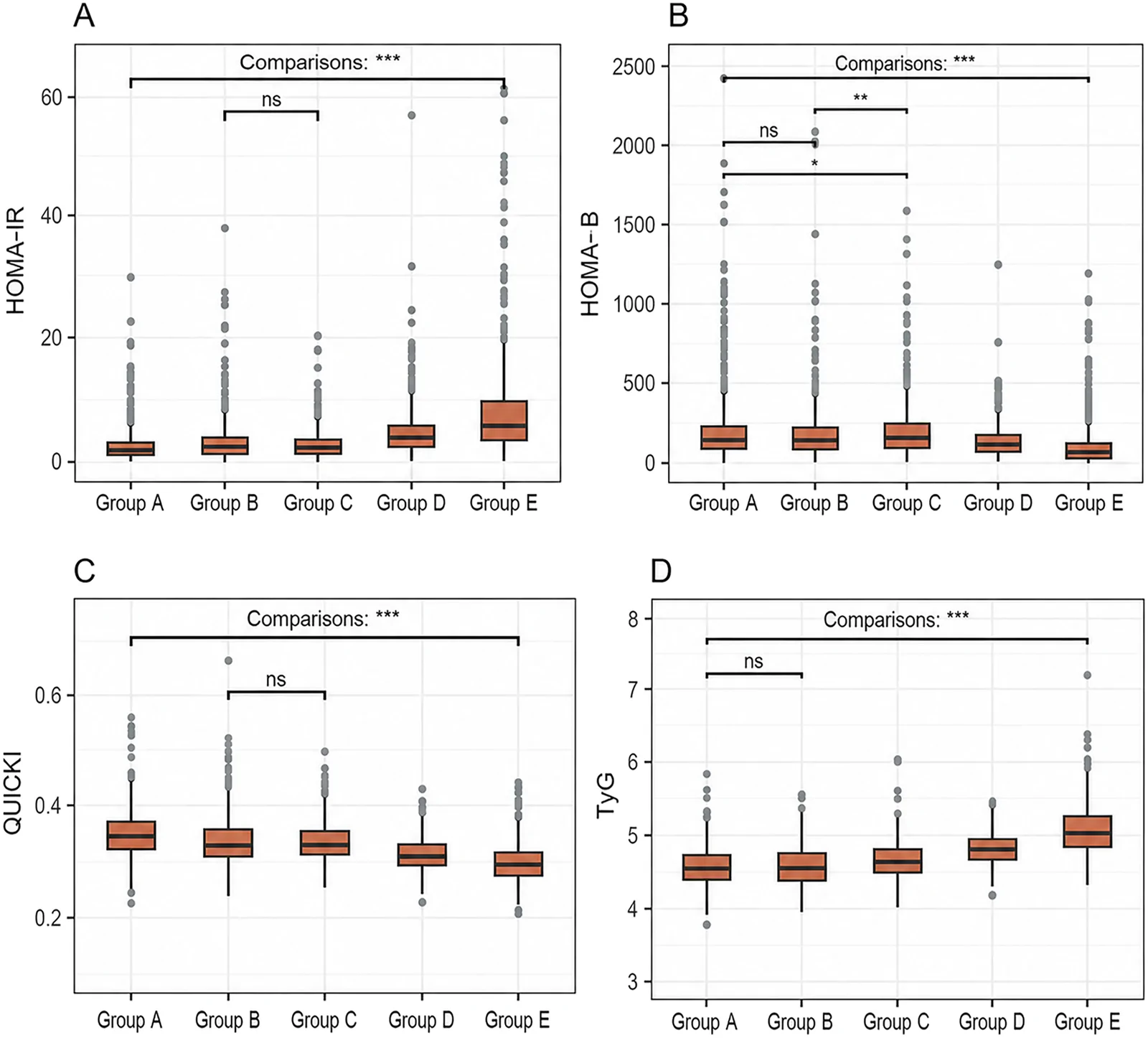
Comparison of insulin resistance and sensitivity indices across study groups. Boxplots represent the distribution of HOMA-IR (A), HOMA-B (B), QUICKI (C), and the triglyceride-to-glucose ratio (TyG, D) across five groups. Boxes represent median and IQR; p-values correspond to Bonferroni-adjusted post-hoc comparisons after Kruskal–Wallis testing. ns=not significant; *p<0.05; **p<0.01; ***p<0.001.

HOMA-IR (Figure 3A) revealed highly significant differences between groups (p<0.001), except between Groups B and C. Group B had significantly higher values than Groups A and C (p<0.001). HOMA-β (Figure 3B) followed a descending trend starting from Group C. Group B had lower values than Group C (p<0.01), though still higher than Groups D and E (p<0.001). No significant differences were found between Groups A and B. QUICKI (Figure 3C) progressively decreased from Group A to E (p<0.001), with the exception of the B vs. C comparison. TyG (Figure 3D) increased significantly across groups (p<0.001), except between Groups A and B.

#### Inflammatory markers

**Figure 4:**
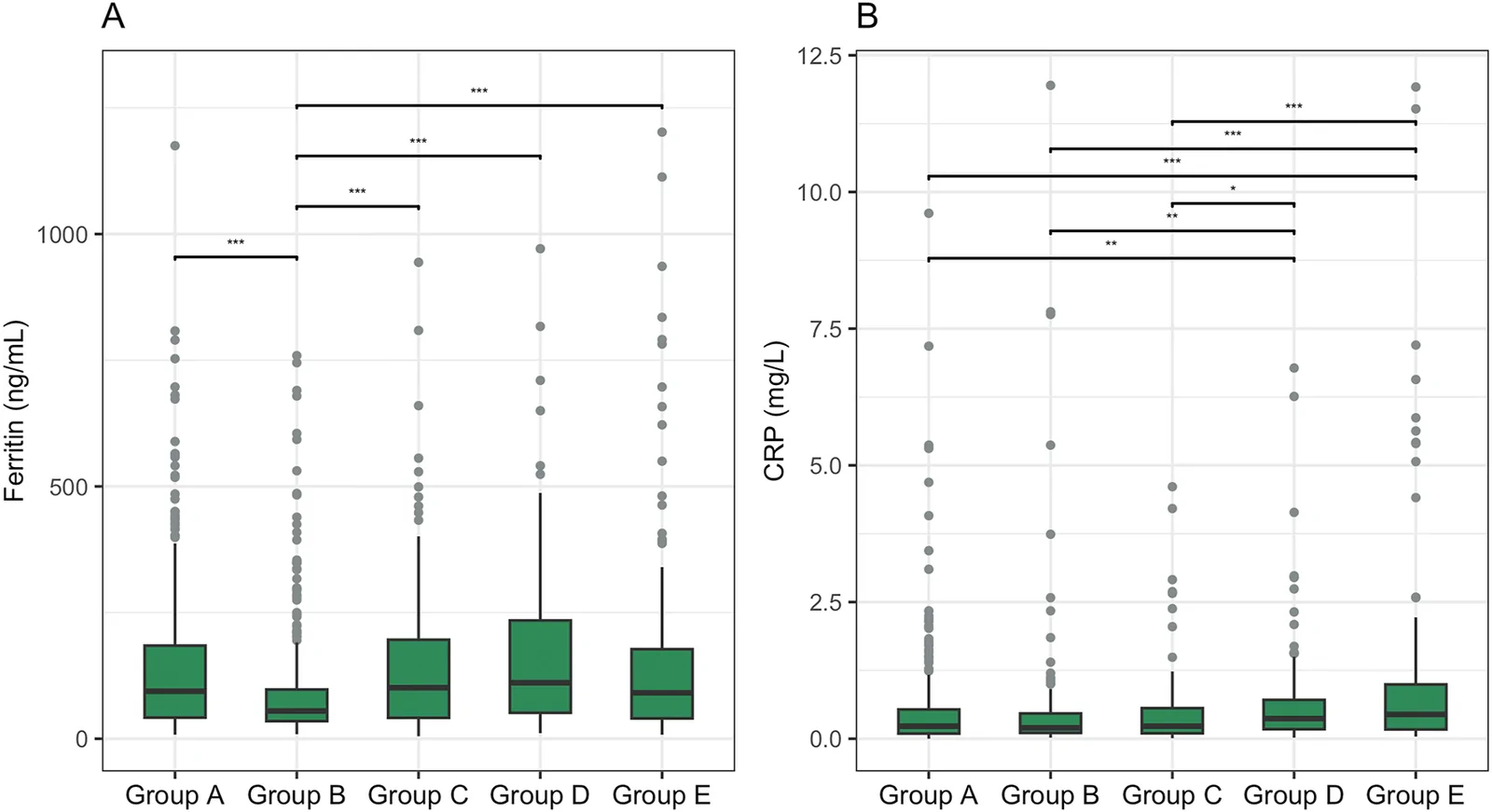
Distribution of inflammatory biomarkers across study groups. Boxplots display serum ferritin levels (A) and C-reactive protein (CRP) concentrations (B) across five groups. Boxes represent median and IQR; p‐values correspond to Bonferroni-adjusted post-hoc comparisons after Kruskal–Wallis testing.*p<0.05; **p<0.01; ***p<0.001.

Ferritin levels (Figure 4A) were significantly higher in Groups A, C, D, and E compared with Group B (p<0.001), with no significant differences between other groups.

Regarding CRP levels (Figure 4B), Group D had higher values than Groups C (p<0.05), A, and B (p<0.001), while Group E exceeded Groups A, B, and C (p<0.0001). No differences were found between Groups A–C or between Groups D and E.

#### Sex-based differences

Sex-stratified analyses revealed consistently higher fibrosis index values in males across most glycaemic categories, particularly for APRI, FIB-4, m-APRI, and Forns scores. Insulin resistance indices also tended to be higher in males, especially HOMA-IR and TyG, whereas QUICKI values were correspondingly lower. Differences in HOMA-β were less consistent across groups (Supplementary Figures 1 and 2; Supplementary Table 3).

### Predictive analysis

**Figure 5:**
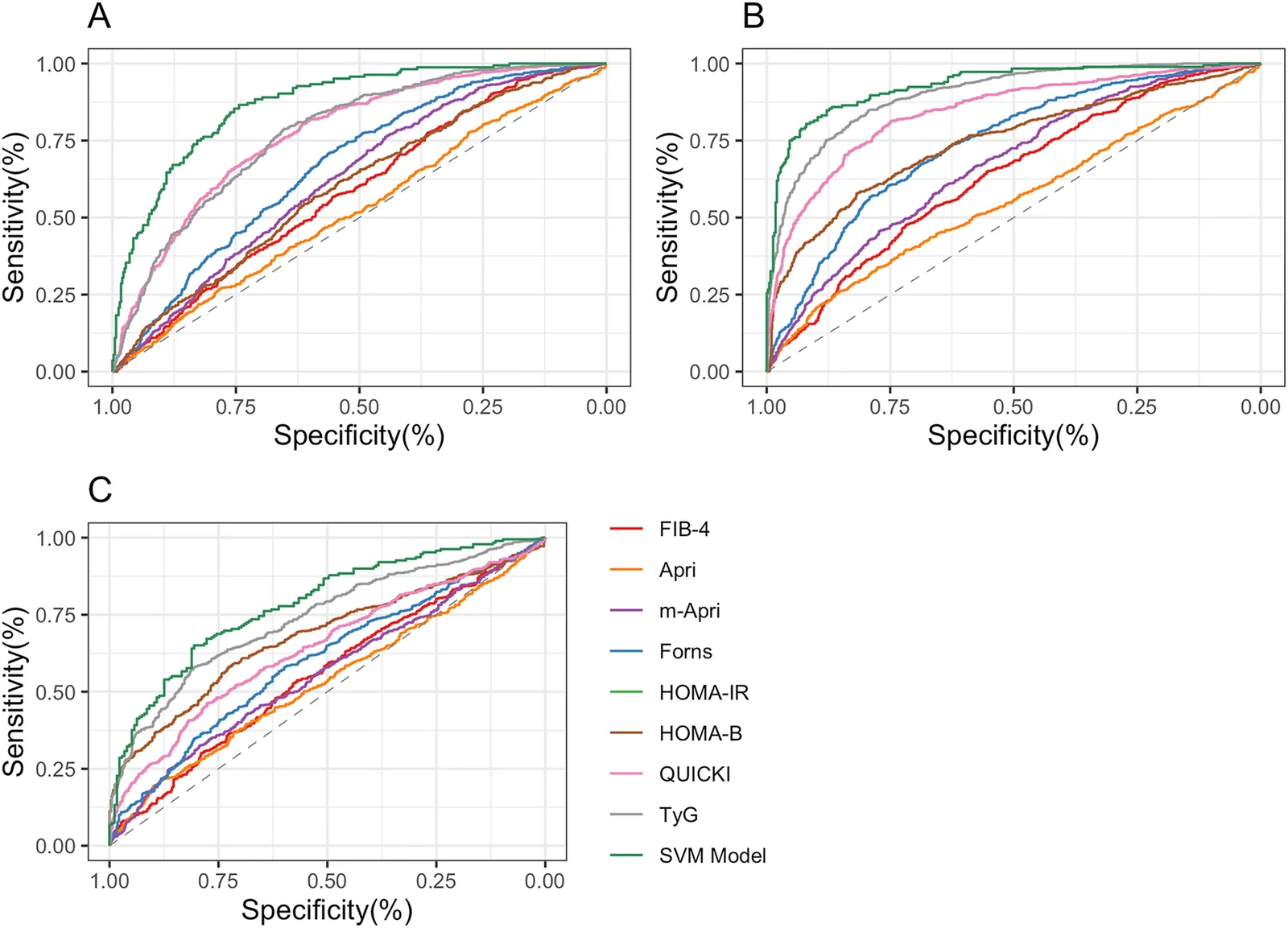
ROC curve analysis comparing discriminatory performance between study groups. The SVM model was trained using the best-performing fibrosis and insulin resistance index for each pairwise comparison. (A) Group A vs. Group D; (B) Group A vs. Group E; (C) Group D vs. Group E.

In the comparison between Groups A and D, the highest AUCs were observed for QUICKI (AUC=0.78; 95 % CI: 0.76–0.80) and TyG (AUC=0.78; 95 % CI: 0.75–0.80), while fibrosis indices such as FIB-4 and APRI demonstrated more modest performance. Among fibrosis scores, the Forns index showed the best discriminative capacity (AUC=0.67; 95 % CI: 0.64–0.69) and was therefore selected for inclusion in the model. The optimized SVM model outperformed all individual markers, achieving an AUC of 0.87 (95 % CI: 0.84–0.90) (Table 1, Figure 5A).

**Table 1:** Comparative performance metrics of individual biomarkers and an optimized SVM model combining top fibrosis and insulin resistance markers across pairwise clinical group comparisons.

Group A vs. Group D							
	AUC (95 %CI)	CO	S	E	PPV	NPV	A
FIB-4	0.59 (0.56–0.61)	0.71	77 %	36 %	36 %	78 %	49 %
APRI	0.53 (0.50–0.55)	0.20	78 %	28 %	33 %	73 %	43 %
m-APRI	0.63 (0.60–0.65)	24.47	77 %	44 %	39 %	81 %	54 %
Forns^a^	0.67 (0.64–0.69)	36.63	73 %	54 %	43 %	81 %	60 %
HOMA-IR	0.77 (0.75–0.80)	23.87	80 %	61 %	49 %	87 %	67 %
HOMA-B	0.60 (0.57–0.63)	127.42	57 %	59 %	39 %	75 %	58 %
QUICKI^a^	0.78 (0.76–0.80)	0.33	80 %	61 %	49 %	87 %	67 %
TyG	0.78 (0.75–0.80)	46.42	79 %	65 %	51 %	87 %	69 %
SVM model	0.87 (0.84–0.90)	0.28	87 %	74 %	58 %	93 %	78 %

**Group A vs. Group E**							

	**AUC (95 %CI)**	**CO**	**S**	**E**	**PPV**	**NPV**	**A**

FIB-4	0.63 (0.61–0.66)	0.94	65 %	55 %	41 %	77 %	58 %
APRI	0.56 (0.53–0.58)	0.43	26 %	85 %	45 %	71 %	66 %
m-APRI	0.67 (0.65–0.70)	31.03	66 %	58 %	43 %	79 %	61 %
Forns^a^	0.74 (0.72–0.76)	40.69	73 %	63 %	49 %	83 %	66 %
HOMA-IR	0.84 (0.82–0.86)	31.71	81 %	75 %	60 %	90 %	77 %
HOMA-B	0.74 (0.72–0.77)	815.85	58 %	82 %	60 %	80 %	74 %
QUICKI	0.84 (0.82–0.86)	0.33	80 %	61 %	49 %	87 %	67 %
TyG^a^	0.90 (0.89–0.91)	47.64	85 %	79 %	66 %	92 %	81 %
SVM model	0.93 (0.91–0.95)	0.30	85 %	87 %	76 %	93 %	87 %

**Group D vs. Group E**							

	**AUC (95 %CI)**	**CO**	**S**	**E**	**PPV**	**NPV**	**A**

FIB-4	0.56 (0.52–0.59)	1.10	54 %	57 %	56 %	55 %	55 %
APRI	0.53 (0.50–0.57)	0.52	19 %	90 %	66 %	52 %	54 %
m-APRI	0.56 (0.53–0.59)	53.99	33 %	79 %	62 %	53 %	56 %
Forns^a^	0.60 (0.57–0.63)	48.01	58 %	60 %	60 %	58 %	59 %
HOMA-IR	0.64 (0.61–0.67)	6.36	46 %	78 %	68 %	58 %	62 %
HOMA-B	0.68 (0.65–0.71)	823.05	58 %	73 %	69 %	63 %	65 %
QUICKI	0.64 (0.61–0.67)	0.29	46 %	78 %	68 %	58 %	62 %
TyG^a^	0.74 (0.71–0.77)	49.88	58 %	81 %	75 %	65 %	69 %
SVM model	0.78 (0.74–0.83)	0.52	65 %	81 %	78 %	68 %	73 %

A, overall accuracy; CO, optimal cut off; Chol, cholesterol; E, specificity; NPV, negative predictive value; PPV, positive predictive value; S, sensitivity; SVM, support vector machine; TG, triglycerides. ^a^Parameters included in the SVM model.

In the Groups A vs. E comparison, TyG yielded the highest individual performance (AUC=0.90; 95 % CI: 0.89–0.91). Among fibrosis indices, the Forns index again displayed reasonable discriminative ability (AUC=0.74; 95 % CI: 0.72–0.76). The SVM model again outperformed all individual metrics, achieving an AUC of 0.93 (95 % CI: 0.91–0.95) (Table 1, Figure 5B).

In the comparison between Groups D and E, TyG remained the top-performing individual biomarker (AUC=0.74; 95 % CI: 0.71–0.77), whereas fibrosis indices showed lower discriminative performance. The Forns index again performed best among them (AUC=0.60; 95 % CI: 0.57–0.63). The SVM model demonstrated superior performance, achieving an AUC of 0.78 (95 % CI: 0.74–0.83) (Table 1, Figure 5C).

In all SVM models, the linear kernel consistently achieved the highest performance (Supplementary Table 4) and was therefore used throughout this section.

## Discussion

The frequent coexistence of T2DM and MASLD reflects the convergence of shared pathophysiological mechanisms dominated by insulin resistance and chronic low-grade metabolic inflammation, which together promote the simultaneous development of glycaemic, lipid, and hepatic alterations [23], 24]. Within this framework, the present study evaluated the combined utility of surrogate indices of insulin resistance and non-invasive liver fibrosis scores for hepatometabolic stratification across the dysglycaemia spectrum using routinely available laboratory parameters in a real-world clinical setting. In this context, the present results support the interpretation of routine laboratory-derived indices as complementary markers of hepatometabolic interaction rather than isolated surrogates of insulin resistance or fibrosis severity.

Correlation analyses provided the main interpretative structure for integrating the group-level findings. Overall, the results support the concept that insulin resistance, dysglycaemia, dyslipidaemia, and early liver injury represent interconnected manifestations of a common metabolic axis characterized by subclinical inflammation and systemic metabolic dysfunction, consistent with previous mechanistic models proposed by Stefan et al. [25]. These findings reinforce the notion that hepatometabolic alterations do not develop as isolated processes but rather evolve along coordinated trajectories linking metabolic signalling disturbances with progressive hepatic involvement.

Within this shared framework, insulin resistance indices showed consistent associations with glycaemic, lipid, hepatic, and inflammatory parameters, supporting their role as early integrators of hepatometabolic deterioration. In particular, the positive correlations observed for HOMA-IR and TyG, together with the inverse correlations for QUICKI, reinforce the hypothesis that compensatory hyperinsulinaemia promotes persistent hepatic gluconeogenesis, enhanced *de novo* lipogenesis, and adipose tissue dysfunction, thereby contributing to a lipotoxic and pro-inflammatory environment that favours the transition from metabolic dysregulation to structural liver injury [25], 26]. Importantly, the central position of these indices within the correlation structure observed in the present study further supports their utility as laboratory-accessible indicators of early metabolic stress preceding overt hepatic damage. The limited magnitude of direct correlations observed between insulin resistance indices and fibrosis scores further suggests that the transition from metabolic dysfunction to structural hepatic involvement may be mediated by intermediate inflammatory and metabolic processes rather than following a simple linear trajectory.

Among the evaluated fibrosis scores, the Forns index was the only marker simultaneously associated with glycaemic, lipid, inflammatory, and insulin-resistance parameters, particularly TyG. This behaviour suggests a closer integration with the underlying pathophysiological processes linking metabolic dysfunction and early hepatic injury. The inclusion of GGT, total cholesterol, and platelet count in its formulation may explain this broader metabolic sensitivity, as these variables capture complementary aspects of oxidative stress, lipid metabolism, and early portal haemodynamic alterations that are not simultaneously reflected in other fibrosis indices. In contrast to FIB-4 and m-APRI, which are primarily oriented toward identifying more advanced stages of fibrosis, the Forns index may therefore reflect earlier hepatometabolic interactions occurring before clinically overt structural liver disease becomes established.

Against this mechanistic background, the progressive distribution of insulin resistance and fibrosis indices across glycaemic stages further supports the existence of a structured hepatometabolic continuum. A parallel increase in HOMA-IR, TyG, FIB-4, m-APRI, Forns index, and CRP from intermediate dysglycaemic stages to established T2DM suggests that insulin resistance may act as a central driver of the transition from subclinical metabolic dysfunction to structural hepatic involvement. These findings are consistent with previous studies demonstrating that MASLD promotes glucose intolerance progression [27], 28], that hepatic fibrosis advances more rapidly in diabetic than in non-diabetic individuals [29], and that increasing HbA_1c_ levels are independently associated with greater fibrotic burden in MASLD [30]. The progressive elevation of CRP observed in more advanced dysglycaemic stages further supports the contribution of systemic inflammation to early hepatometabolic deterioration, even before overt diabetes develops [31]. Experimental evidence indicating that early alterations in intracellular metabolic signalling precede clinically detectable insulin resistance further reinforces the rationale for biomarker-based screening strategies aimed at identifying reversible stages of metabolic progression [32].

From a discriminative perspective, the TyG index showed the highest individual performance for identifying progressive metabolic impairment, confirming its value as an integrated marker of insulin resistance and lipid dysfunction. Among fibrosis indices, the Forns index consistently demonstrated the strongest discriminative capacity and the broadest metabolic correlations. The selection of these indices for SVM-based classification modelling was therefore consistent with their central role within the observed hepatometabolic correlation network. Importantly, the integration of these indices through support vector machine models improved classification performance compared with individual markers alone, suggesting that the combined interpretation of routinely available laboratory-derived indices may provide a complementary framework for hepatometabolic stratification beyond conventional biochemical parameters.

Notably, all variables included in both the individual indices and the composite models derive from routine laboratory testing and do not require additional sampling or specialised techniques. Their potential implementation within laboratory information systems could therefore enable opportunistic identification of complex metabolic profiles associated with dysglycaemia in routine clinical workflows, particularly in primary care settings, supporting earlier targeted clinical evaluation in a scenario of increasing metabolic disease burden and reinforcing the emerging role of the clinical laboratory as an active component in metabolic risk stratification strategies based on routinely generated analytical data.

Several limitations should be considered. First, the cross-sectional design and the absence of validation against reference methods such as liver elastography or histological assessment require cautious interpretation of the findings regarding structural liver involvement. Second, the absence of relevant clinical variables – including body mass index, visceral adiposity distribution, pharmacological treatments, and hepatic imaging findings – may have influenced the observed associations. Third, inclusion required fasting insulin measurements, which may have introduced selection bias toward individuals undergoing metabolic evaluation. Fourth, because glycaemic groups were defined using fasting glucose and HbA_1c_ and some evaluated indices include glucose-related variables, partial incorporation bias cannot be excluded; therefore, classification models should be interpreted as tools for characterizing hepatometabolic profiles across dysglycaemic stages rather than as independent diagnostic models. Finally, the lack of longitudinal follow-up precludes causal inference and limits the development of predictive models of metabolic progression.

## Conclusions

Our findings support the combined use of surrogate indices of insulin resistance and laboratory-derived fibrosis scores – particularly TyG and the Forns index – as accessible tools for hepatometabolic stratification across the dysglycaemia spectrum. Their integration through machine-learning approaches using routine laboratory data may enable opportunistic identification of early metabolic deterioration and support laboratory-driven risk stratification strategies in real-world clinical settings.

## Supplementary Material

Supplementary Material

Supplementary Material

Supplementary Material

Supplementary Material

Supplementary Material

Supplementary Material
