# Supplementary material for: Routine biochemical indices for hepatometabolic stratification across the dysglycaemia spectrum: insulin resistance, fibrosis scores, and machine-learning integration
Source: Adv Lab Med. 2026 Jul 14;7(3):211–21. doi: 10.1515/almed-2026-0066 (PMC13552813; doi:10.1515/almed-2026-0066)
Supplement: Supplementary file 1 [file j_almed-2026-0066_suppl_001.docx]

**Supplementary Table 1. Instrumental methodologies for the different biomarkers**

| **Biomarker** | **Methodology** |
| --- | --- |
| Albumin | Colorimetry |
| ALT | UV kinetics |
| AST | UV kinetics |
| Chol | Enzymatic colorimetry |
| CRP | Immunoturbidimetry |
| Ferritin | Immunoturbidimetry |
| GGT | Enzymatic colorimetry |
| Glu | UV enzymatic |
| HDL | Enzymatic colorimetry |
| HbA1c | High-Performance Liquid Chromatography |
| Insulin | Chemiluminescence |
| LDL | Enzymatic colorimetry |
| Platelet | Electrical impedance |
| Proteins | Colorimetry |
| TG | Enzymatic colorimetry |

ALT: Alanine aminotransferase; AST: Aspartate aminotransferase; Chol: Cholesterol; CRP: C-Reactive Protein; GGT: Gamma-glutamyl transferase; Glu: Fasting Glucose; HDL: HDL Cholesterol; HbA1c: Glycated hemoglobin; LDL: LDL Cholesterol; TG: Triglycerides; UV: ultraviolet.
