# Supplementary material for: Routine biochemical indices for hepatometabolic stratification across the dysglycaemia spectrum: insulin resistance, fibrosis scores, and machine-learning integration
Source: Adv Lab Med. 2026 Jul 14;7(3):211–21. doi: 10.1515/almed-2026-0066 (PMC13552813; doi:10.1515/almed-2026-0066)
Supplement: Supplementary file 2 [file j_almed-2026-0066_suppl_002.docx]

**Supplementary Table 2.- Descriptive Statistics of Clinical and Biochemical Parameters by Patient Group.**

|  | **Group A (n)** | **Median (IQR)** | **Group B (n)** | **Median (IQR)** | **Group C (n)** | **Median (IQR)** | **Group D (n)** | **Median (IQR)** | **Group E (n)** | **Median (IQR)** |
| --- | --- | --- | --- | --- | --- | --- | --- | --- | --- | --- |
| **Age** | 653 | 45 (20) | 378 | 50 (21) | 305 | 51 (19) | 310 | 55 (15) | 303 | 58 (16.5) |
| **ALT** | 653 | 20 (15) | 375 | 21 (12) | 305 | 22 (14) | 310 | 27 (20) | 303 | 27 (22) |
| **APRI** | 649 | 0.26 (0.17) | 364 | 0.27 (0.14) | 305 | 0.26 (0.19) | 310 | 0.28 (0.19) | 303 | 0.29 (0.24) |
| **AST/ALT** | 651 | 1.12 (0.54) | 365 | 1.18 (0.66) | 305 | 1 (0.48) | 310 | 0.89 (0.38) | 303 | 0.92 (0.39) |
| **Albumin** | 653 | 4.2 (0.4) | 378 | 4.5 (0.5) | 305 | 4.2 (0.4) | 310 | 4.2 (0.4) | 303 | 4.2 (0.4) |
| **Chol** | 653 | 187 (53) | 373 | 181 (50) | 305 | 193 (53) | 310 | 192 (48.25) | 303 | 175 (56) |
| **Chol/HDL** | 653 | 3.58 (1.23) | 373 | 3.61 (1.06) | 305 | 3.82 (1.4) | 310 | 3.89 (1.29) | 303 | 3.92 (1.49) |
| **FIB-4** | 649 | 0.88 (0.63) | 364 | 0.35 (0.76) | 305 | 0.95 (0.69) | 310 | 1 (0.69) | 303 | 1.17 (0.78) |
| **Forns** | 638 | 3.44 (2.43) | 117 | 4.7 (2.42) | 305 | 3.87 (2.36) | 310 | 4.41 (1.94) | 303 | 5.15 (2.12) |
| **GGT** | 642 | 22 (20) | 133 | 31 (35) | 305 | 27 (23) | 310 | 33 (29) | 303 | 35 (36.5) |
| **Glu** | 653 | 87 (12) | 378 | 102 (26) | 305 | 90 (10) | 310 | 108 (10.75) | 303 | 143 (51.5) |
| **HDL** | 653 | 51 (17) | 378 | 49.5 (15) | 305 | 50 (18) | 310 | 48.5 (15) | 303 | 44 (14) |
| **HOMA-B** | 646 | 150.65 (138.9) | 356 | 156.82 (133.97) | 305 | 166.24 (151.43) | 310 | 123.71 (114.11) | 303 | 72 (91.61) |
| **HOMA-IR** | 653 | 1.95 (2.12) | 371 | 2.9 (3.09) | 305 | 2.74 (2.2) | 310 | 4.26 (3.65) | 303 | 6.36 (6.68) |
| **HbA1c** | 653 | 5.4 (0.3) | 378 | 5.4 (0.4) | 305 | 5.8 (0.3) | 310 | 6 (0.4) | 303 | 7.2 (1.6) |
| **Insulin** | 653 | 9.2 (8.6) | 371 | 12.5 (12.65) | 305 | 12.4 (10.3) | 310 | 15.8 (13.5) | 303 | 16.9 (15.4) |
| **LDLc** | 635 | 111 (42) | 366 | 106 (42) | 299 | 114 (45.5) | 292 | 115 (41.75) | 275 | 98 (45.5) |
| **MEG** | 649 | 108.28 (8.61) | 356 | 111.15 (8.61) | 305 | 119.76 (8.61) | 310 | 125.5 (11.48) | 303 | 159.94 (45.92) |
| **Platelet** | 651 | 241 (86) | 377 | 260 (97) | 305 | 242 (101) | 310 | 247.5 (85.5) | 303 | 237 (86) |
| **Proteins** | 558 | 7 (0.6) | 221 | 7.3 (0.6) | 274 | 7 (0.6) | 266 | 7.1 (0.6) | 265 | 7.1 (0.7) |
| **QUICKI** | 649 | 0.35 (0.05) | 356 | 0.32 (0.05) | 305 | 0.33 (0.04) | 310 | 0.31 (0.04) | 303 | 0.29 (0.04) |
| **TG** | 653 | 103 (70) | 378 | 97 (65) | 305 | 119 (72) | 310 | 132 (73) | 303 | 163 (95) |
| **TG/Chol** | 653 | 4.54 (0.33) | 378 | 4.55 (0.38) | 305 | 4.64 (0.32) | 310 | 4.79 (0.29) | 303 | 5.03 (0.39) |
| **TG/HDL** | 653 | 2.02 (1.73) | 378 | 2.02 (1.67) | 305 | 2.41 (2.11) | 310 | 2.71 (2.19) | 303 | 3.74 (2.56) |
| **m-APRI** | 649 | 2.7 (2.33) | 364 | 1.04 (2.7) | 305 | 3.11 (2.63) | 310 | 3.63 (2.61) | 303 | 3.97 (3.7) |
| **non-HDL** | 653 | 136 (48) | 373 | 129 (47) | 305 | 140 (49) | 310 | 141 (45.75) | 303 | 129 (52) |

ALT: Alanine aminotransferase; AST: Aspartate aminotransferase; Chol: Cholesterol; GGT: Gamma-glutamyl transferase; Glu: Fasting glucose; HbA1c: Glycated hemoglobin; TG: Triglycerides.
