# Supplementary material for: Routine biochemical indices for hepatometabolic stratification across the dysglycaemia spectrum: insulin resistance, fibrosis scores, and machine-learning integration
Source: Adv Lab Med. 2026 Jul 14;7(3):211–21. doi: 10.1515/almed-2026-0066 (PMC13552813; doi:10.1515/almed-2026-0066)
Supplement: Supplementary file 3 [file j_almed-2026-0066_suppl_003.docx]

**Supplementary Table 3. Median and Interquartile Ranges of Age and Biomarker Values by Clinical Group and Sex.**

|  | **Group A** | | | | **Group B** | | | | **Group C** | | | | **Group D** | | | | **Group E** | | | |
| --- | --- | --- | --- | --- | --- | --- | --- | --- | --- | --- | --- | --- | --- | --- | --- | --- | --- | --- | --- | --- |
|  | **Male** | | **Female** | | **Male** | | **Female** | | **Male** | | **Female** | | **Male** | | **Female** | | **Male** | | **Female** | |
|  | **n** | **Median (IQR)** | **n** | **Median (IQR)** | **n** | **Median (IQR)** | **n** | **Median (IQR)** | **n** | **Median (IQR)** | **n** | **Median (IQR)** | **n** | **Median (IQR)** | **n** | **Median (IQR)** | **n** | **Median (IQR)** | **n** | **Median (IQR)** |
| **Age** | 218 | 47(19) | 435 | 43(19) | 175 | 51 (19) | 203 | 49 (19) | 102 | 47.50(17.75) | 203 | 52(19) | 139 | 56(17) | 171 | 54(15.50) | 151 | 58(16) | 152 | 59(17) |
| **ALT** | 218 | 25(18.75) | 435 | 17(12) | 173 | 22(12) | 202 | 19(11.75) | 102 | 28(18.75) | 203 | 20(10) | 139 | 34(18) | 171 | 23(19) | 151 | 31(26) | 152 | 23.50(18.25) |
| **APRI** | 216 | 0.31(0.20) | 433 | 0.24(0.14) | 168 | 0.29(0.16) | 196 | 0.26(0.13) | 102 | 0.33(0.19) | 203 | 0.23(0.15) | 139 | 0.32(0.21) | 171 | 0.24(0.14) | 151 | 0.33(0.28) | 152 | 0.24(0.18) |
| **AST/ALT** | 217 | 0.96(0.52) | 434 | 1.19(0.56) | 169 | 1.12(0.58) | 196 | 1.26(0.71) | 102 | 0.88(0.46) | 203 | 1.08(0.47) | 139 | 0.81(0.31) | 171 | 0.96(0.45) | 151 | 0.83(0.36) | 152 | 1(0.41) |
| **Albumin** | 218 | 4.40(0.50) | 435 | 4.20(0.30) | 175 | 4.50(0.40) | 203 | 4.40(0.40) | 102 | 4.35(0.40) | 203 | 4.10(0.30) | 139 | 4.30(0.40) | 171 | 4.20(0.30) | 151 | 4.30(0.40) | 152 | 4.10(0.40) |
| **Chol** | 218 | 182.50(48) | 435 | 189(52) | 173 | 176(48) | 200 | 184.50(50) | 102 | 188.50(50) | 203 | 194(52.50) | 139 | 186(55.50) | 171 | 195(45) | 151 | 167(56) | 152 | 183(51.25) |
| **Chol/HDL** | 218 | 3.90(1.39) | 435 | 3.45(1.11) | 173 | 3.66(1.03) | 200 | 3.57(1.05) | 102 | 4.20(1.31) | 203 | 3.52(1.22) | 139 | 4.12(1.10) | 171 | 3.75(1.30) | 151 | 4.09(1.62) | 152 | 3.79(1.30) |
| **FIB.4** | 216 | 1.03(0.68) | 433 | 0.82(0.59) | 168 | 0.33(0.82) | 196 | 0.37(0.69) | 102 | 1(0.72) | 203 | 0.92(0.69) | 139 | 1.08(0.74) | 171 | 0.94(0.58) | 151 | 1.23(0.81) | 152 | 1.03(0.82) |
| **Forns** | 207 | 4.08(1.97) | 431 | 3.04(2.23) | 58 | 5.31(1.75) | 59 | 3.71(2.43) | 102 | 4.37(2.24) | 203 | 3.72(2.18) | 139 | 4.75(2.02) | 171 | 4.05(1.96) | 151 | 5.46(1.62) | 152 | 4.69(2.28) |
| **GGT** | 209 | 32(27) | 433 | 19(14) | 64 | 41(42) | 69 | 26(25) | 102 | 36(31.25) | 203 | 22(16) | 139 | 38(28) | 171 | 29(26.50) | 151 | 40(35) | 152 | 34(30.75) |
| **Glu** | 218 | 87.50(11.75) | 435 | 87(13) | 175 | 107 (19) | 203 | 102 (21) | 102 | 92.50(9.75) | 203 | 90(11) | 139 | 108(11) | 171 | 108(10) | 151 | 144(52) | 152 | 141(48.25) |
| **HDL** | 218 | 46(14) | 435 | 54(17.50) | 175 | 48(12) | 203 | 51(17) | 102 | 43(13.75) | 203 | 54(18.50) | 139 | 44(14.50) | 171 | 51(15) | 151 | 41(12.50) | 152 | 46(12.25) |
| **HOMA.B** | 216 | 150.98(149.39) | 430 | 150.27(132.75) | 167 | 157.76(132.68) | 189 | 152.47(133.26) | 102 | 172.36(151.56) | 203 | 162(146.44) | 139 | 126.46(123.70) | 171 | 119.70(106.04) | 151 | 68.77(83.22) | 152 | 80.86(108.58) |
| **HOMA.IR** | 218 | 2.22(2.49) | 435 | 1.89(1.87) | 174 | 3.14(2.94) | 197 | 2.57(3.25) | 102 | 3.20(2.58) | 203 | 2.60(2.13) | 139 | 4.28(4.79) | 171 | 4.24(3.50) | 151 | 6.07(7.13) | 152 | 6.67(6.27) |
| **HbA1c** | 218 | 5.40(0.30) | 435 | 5.40(0.30) | 175 | 5.40(0.40) | 203 | 5.40(0.30) | 102 | 5.80(0.30) | 203 | 5.80(0.30) | 139 | 6(0.40) | 171 | 6(0.40) | 151 | 7.20(1.60) | 152 | 7.20(1.60) |
| **Insulin** | 218 | 9.80(11.55) | 435 | 8.80(8) | 174 | 13.40(11.97) | 197 | 11.50(13) | 102 | 13.80(10.80) | 203 | 12(9.75) | 139 | 16.40(17.30) | 171 | 15.60(12.75) | 151 | 16.40(15.50) | 152 | 18.60(15.50) |
| **LDLc** | 211 | 109(40) | 424 | 112.50(43.25) | 167 | 104(35.50) | 199 | 107(47.50) | 100 | 116.50(50.25) | 199 | 113(43.50) | 128 | 110.50(46) | 164 | 116(38.25) | 133 | 91(50) | 142 | 101.50(43.25) |
| **MEG** | 216 | 106.84(8.61) | 433 | 108.28(8.61) | 167 | 111.15 (8.61) | 189 | 111.15 (8.61) | 102 | 119.76(8.61) | 203 | 119.76(8.61) | 139 | 125.50(11.48) | 171 | 125.50(11.48) | 151 | 159.94(45.92) | 152 | 159.94(45.92) |
| **Platelet** | 217 | 217(72) | 434 | 256(93.75) | 174 | 251(98.50) | 203 | 268(87) | 102 | 223(71.50) | 203 | 264(110.50) | 139 | 231(88.50) | 171 | 264(84) | 151 | 221(84) | 152 | 254.50(98.50) |
| **Proteins** | 183 | 7.20(0.60) | 375 | 7(0.60) | 105 | 7.40(0.60) | 116 | 7.30(0.60) | 86 | 7.20(0.60) | 188 | 7(0.60) | 115 | 7.10(0.55) | 151 | 7.10(0.80) | 130 | 7.15(0.70) | 135 | 7(0.60) |
| **QUICKI** | 216 | 0.34(0.05) | 433 | 0.35(0.05) | 167 | 0.32(0.04) | 189 | 0.33(0.06) | 102 | 0.32(0.04) | 203 | 0.33(0.04) | 139 | 0.31(0.04) | 171 | 0.31(0.04) | 151 | 0.29(0.04) | 152 | 0.29(0.04) |
| **TG** | 218 | 110(77.25) | 435 | 99(63) | 175 | 101(67.50) | 203 | 93(61.50) | 102 | 121(73.50) | 203 | 119(68.50) | 139 | 132(74.50) | 171 | 132(73) | 151 | 168(107) | 152 | 160(96.50) |
| **TG/Chol** | 218 | 4.61(0.35) | 435 | 4.52(0.31) | 175 | 4.60(0.37) | 203 | 4.52(0.39) | 102 | 4.66(0.33) | 203 | 4.63(0.31) | 139 | 4.79(0.29) | 171 | 4.79(0.28) | 151 | 5.05(0.38) | 152 | 5.02(0.41) |
| **TG/HDL** | 218 | 2.41(2.22) | 435 | 1.78(1.46) | 175 | 2.08(1.62) | 203 | 1.88(1.49) | 102 | 2.96(2.31) | 203 | 2.29(1.82) | 139 | 2.82(2.22) | 171 | 2.62(2.23) | 151 | 3.92(2.91) | 152 | 3.58(2.31) |
| **m.APRI** | 216 | 3.46(2.59) | 433 | 2.35(2.17) | 168 | 1.06(3.50) | 196 | 1.04(2.20) | 102 | 3.68(2.45) | 203 | 2.76(2.54) | 139 | 3.94(2.85) | 171 | 3.17(2.54) | 151 | 4.51(3.88) | 152 | 3.56(3.21) |
| **non-HDL** | 218 | 136(46) | 435 | 135(48) | 173 | 123(44) | 200 | 133(47.75) | 102 | 143.50(53.50) | 203 | 138(45) | 139 | 136(50) | 171 | 143(39) | 151 | 127(55) | 152 | 131(47.25) |

ALT: Alanine aminotransferase; AST: Aspartate aminotransferase; Chol: Cholesterol; GGT: Gamma-glutamyl transferase; FG: Fasting glucose; HbA1c: Glycated hemoglobin; TP: Total proteins; TG: Triglycerides.
