# Supplementary material for: Routine biochemical indices for hepatometabolic stratification across the dysglycaemia spectrum: insulin resistance, fibrosis scores, and machine-learning integration
Source: Adv Lab Med. 2026 Jul 14;7(3):211–21. doi: 10.1515/almed-2026-0066 (PMC13552813; doi:10.1515/almed-2026-0066)
Supplement: Supplementary file 4 [file j_almed-2026-0066_suppl_004.docx]

**Supplementary Table 4. Performance Metrics (AUC and 95% CI) of SVM Models Using Linear, Polynomial, Radial, and Sigmoid Kernels for Each Pairwise Cohort Comparison.**

| **Kernel** | **AUC: A vs D** | **AUC: A vs E** | **AUC: D vs E** |
| --- | --- | --- | --- |
| **Linear** | 0,87 (95%CI: 0,84–0,90) | 0,93 (95%CI: 0,91–0,95) | 0,78 (95%CI: 0,74–0,83) |
| **Polynomial** | 0,86 (95%CI: 0,82–0,89) | 0,91 (95%CI: 0,89–0,94) | 0,78 (95%CI: 0,73–0,82) |
| **Radial** | 0,85 (95%CI: 0,82–0,89) | 0,91 (95%CI: 0,88–0,94) | 0,76 (95%CI: 0,71–0,81) |
| **Sigmoid** | 0,70 (95%CI: 0,65–0,74) | 0,88 (95%CI: 0,84–0,91) | 0,67 (95%CI: 0,62–0,73) |
